# Measuring clinical uncertainty and equipoise by applying the agreement study methodology to patient management decisions

**DOI:** 10.1186/s12874-020-01095-8

**Published:** 2020-08-25

**Authors:** Robert Fahed, Tim E. Darsaut, Behzad Farzin, Miguel Chagnon, Jean Raymond

**Affiliations:** 1 Division of Neurology, The Ottawa Hospital, Civic Campus, 1053 Carling Ave, Ottawa, Ontario K1Y 4E9 Canada; 2 Mackenzie Health Sciences Centre, Department of Surgery, Division of Neurosurgery, University of Alberta Hospital, 8440 - 112 Street, Edmonton, Alberta T6G 2B7 Canada; 3 Department of Radiology, Service of Interventional Neuroradiology, Centre Hospitalier Universitaire de Montréal – CHUM, 1000 Saint-Denis street, room D03-5462B, Montreal, QC H2X 0C1 Canada; 4 Department of Mathematics and Statistic, Pavillion André-Aisenstadt, Université de Montréal, PO Box 6218, succursale Centre-ville, Montreal, Quebec H3C 3J7 Canada

**Keywords:** Equipoise, Uncertainty, Randomized trials, Methodology, Clinical decision-making, Agreement, Reliability, Kappa

## Abstract

**Background:**

Clinical uncertainty and equipoise are vague notions that play important roles in contemporary problems of medical care and research, including the design and conduct of pragmatic trials. Our goal was to show how the reliability study methods normally used to assess diagnostic tests can be applied to particular management decisions to measure the degree of uncertainty and equipoise regarding the use of rival management options.

**Methods:**

We first use thrombectomy in acute stroke as an illustrative example of the method we propose. We then review, item by item, how the various design elements of diagnostic reliability studies can be modified in order to measure clinical uncertainty.

**Results:**

The thrombectomy example shows sufficient disagreement and uncertainty to warrant the conduct of additional randomized trials. The general method we propose is that a sufficient number of diverse individual cases sharing a similar clinical problem and covering a wide spectrum of clinical presentations be assembled into a portfolio that is submitted to a variety of clinicians who routinely manage patients with the clinical problem.

**Discussion:**

Clinicians are asked to independently choose one of the predefined management options, which are selected from those that would be compared within a randomized trial that would address the clinical dilemma. Intra-rater agreement can be assessed at a later time with a second evaluation. Various professional judgments concerning individual patients can then be compared and analyzed using kappa statistics or similar methods. Interpretation of results can be facilitated by providing examples or by translating the results into clinically meaningful summary sentences.

**Conclusions:**

Measuring the uncertainty regarding management options for clinical problems may reveal substantial disagreement, provide an empirical foundation for the notion of equipoise, and inform or facilitate the design/conduct of clinical trials to address the clinical dilemma.

## Background

The notion of clinical uncertainty is vague, but it is involved in many problems of contemporary medical care, research and ethics. Clinical medicine has always been concerned with the care of individuals. Yet, reliable knowledge as to what to do for individual patients is not always available. Pragmatic randomized trials (PRCTs) are increasingly integrated into practice to assess whether medical interventions do good or harm [1–3]. Some pragmatic trials, called ‘care trials’, are even designed to ‘guide practice in the presence of uncertainty’. [4] However, it is common, at least since Fried’s Medical Experimentation [5], that randomized allocation of treatment options and individualized care are placed in opposition to one another [5]. According to Fried, randomized allocation ‘deprives the patient of the benefit of (the doctor’s) individual professional judgment in choosing the therapy’. [5] In Freedman’s Equipoise and the Ethics of Clinical Research [6], ‘in the simplest model’, when testing whether treatment B is better or worse than treatment A, ‘it is necessary that the clinical investigator be in a state of genuine uncertainty regarding the comparative merits’ of treatments A and B. Freedman proposed to ‘call this state of uncertainty about the relative merits of A and B ‘equipoise”. But he found personal equipoise ‘conceptually odd and ethically irrelevant’, and proposed ‘clinical equipoise’ as a better candidate to justify RCTs, because it ‘places the emphasis in informing the patient on the honest disagreement among expert clinicians’. [6] Fried’s personal care model which emphasizes individualized decisions is one reason for the separation of medical care from clinical research that requires randomized allocation. This problem remains the object of ongoing controversies concerning some recent comparative effectiveness trials [7–9]. The fear of being deprived of the doctor’s individual judgment is also the most common reason for not participating in clinical trials [10]. But if doctors really knew what to do for their patients with a particular problem, why would anyone conduct a trial? For many authors, including Fried, prospective observational studies of large data bases could solve the difficulty, but the notion of uncertainty resurfaces: this strategy can only study the comparative merits of rival treatments ‘if there is sufficient uncertainty in practice to ensure that similar patients will be managed differently by different physicians’. [11, 12] If ‘clinical uncertainty’, ‘disagreement among expert clinicians’ and ‘equipoise’ are so important to clinical care and research, can they not also be subjected to verification and quantification?

Fried’s personal care model and Freedman’s notions of clinical uncertainty and equipoise rely on clinical judgment, the use of reason to make the ‘right decision’ and choose the ‘right action’ for a particular patient [13]. The question we want to address is: Can ‘the right action’ for a patient be reliably or repeatedly identified?

**Fig. 1:**
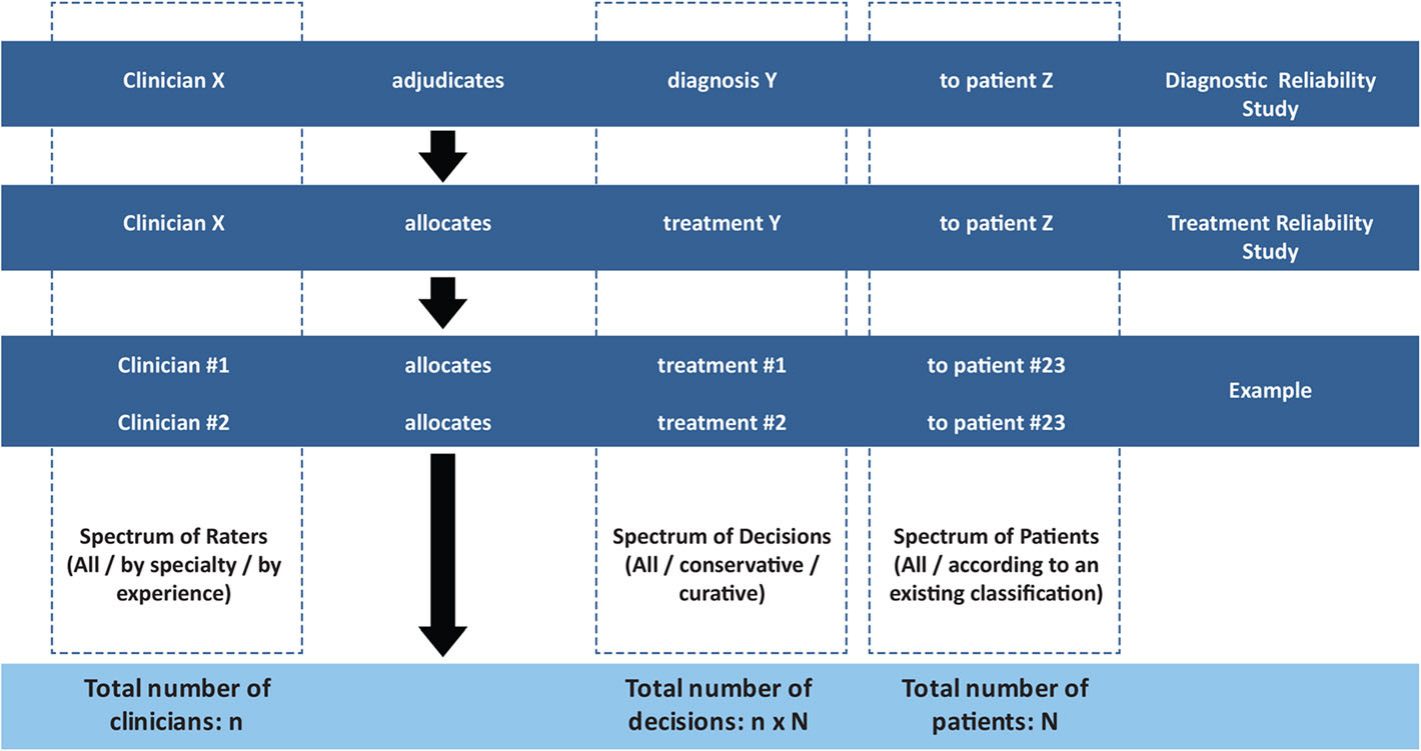
Reliability studies. Reliability studies of diagnostic tests assess the agreement among X clinicians for the diagnosis Y for each of the Z patients included in the study. The reliability studies of treatment decisions we propose use a similar methodology to study the agreement for management options. After asking X clinicians to choose one of the Y management options proposed for each of the Z patients, we can measure the agreement/uncertainty

It seems natural to consider that recommendations made by doctors are inevitably variable and ungeneralizable, for working on a case-by-case basis and following a complex and ‘ineffable’ reasoning process, they take into account the unique histories, characteristics and circumstances of the particular patient [13]. In addition, there has been increasing emphasis on involving patients in shared-decision making, taking into account values and personal preferences [14–16]. This may explain why the reliability of medical recommendations for particular patients has never been tested in medical research (although it is commonly done by patients themselves when they seek a ‘second opinion’). Upon reflection however, the singularity of patients in itself does not make clinicians’ recommendations fundamentally different from other clinical judgments which equally concern unique individuals: the clinician’s verdict (the output of the process) often comes down to allocating the patient to one of a few categories, whether the judgment concerns diagnosis (disease present/absent) or management options (do not treat / treat medically / treat surgically). Treatment decisions or management recommendations made by clinicians are authoritative judgments that have real-life impact on patients. Shouldn’t the reliability of those management decisions be verified? In simple terms, we are more likely to trust the doctor who, when asked the same question twice, provides the same answer both times. No matter the underlying process, if it leads to contradictory judgments or courses of action when the same patient is presented to the same or to different clinicians more than once, then the process is unreliable. The similarities are such that the reliability of clinical recommendations can be assessed the same way the reliability of a diagnostic imaging test is studied (Fig. 1). Furthermore, just as variability in making diagnoses should be studied, rather than minimized or eliminated through consensus sessions, [17] disagreements regarding clinical decisions need not be resolved through multidisciplinary meetings, Delphi processes [18, 19] or practice guidelines unsupported by evidence [20, 21]. Variability and inconsistency in clinical decision making can be informative: the uncertainty can reveal gaps in medical knowledge or identify suboptimal practices that could be improved [22–25]. Measuring the uncertainty in making clinical recommendations can be a preliminary step to the design or conduct of randomized trials [26–30].

We here present an illustrative case [31] and review item by item how we have adapted the standard methodology of reliability/agreement studies normally used for diagnoses to measure clinical uncertainty and equipoise.

### Illustrative example

**Fig. 2:**
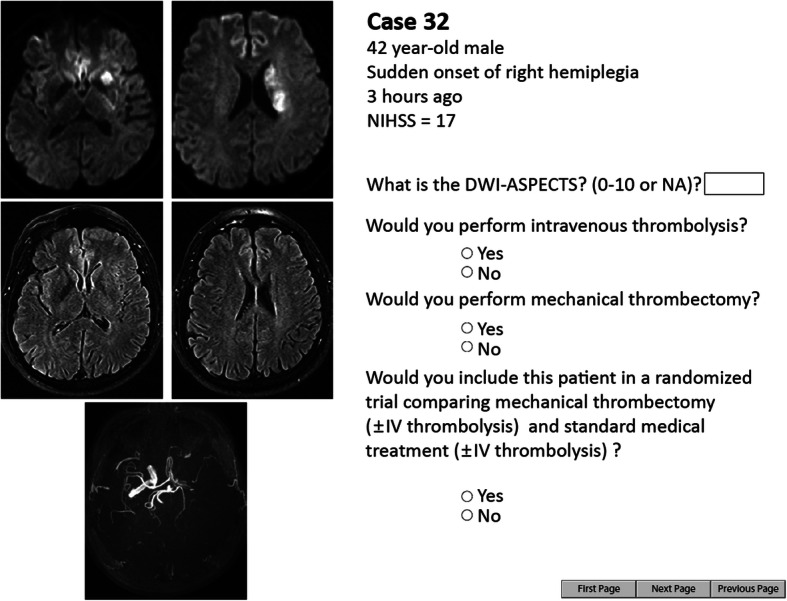
The portfolio. Example from the electronic portfolio used for the thrombectomy agreement study. Each page displayed a clinical vignette with basic clinical information (age, gender, NIHSS score, etc.…) and a few selected brain imaging slices. For each patient, raters were asked whether they would perform mechanical thrombectomy (yes/no). Other questions were also asked for further analyses on other parameters (agreement for intravenous thrombolysis, etc.…)

Thrombectomy, the removal of intracranial clots using intra-arterial catheters, has revolutionized the management of acute stroke from large vessel occlusion [32]. Once the first randomized trial showed improved patient outcomes in October 2014, five other trials were prematurely interrupted, but they still demonstrated the benefits of thrombectomy in studies that included as few as 70 patients [33]. Such large treatment effects were shown because most trials restricted eligibility to most favorable patients, such as early presentation, age < 80, easily accessible thrombus location, and absence of significant cerebral infarction [34]. But how should we care for all other patients? To measure remaining uncertainties regarding the proper use of thrombectomy, a portfolio of 41 patients selected from registries of acute stroke patients was assembled [31]. To obtain a wide spectrum of patients and to balance the frequencies of anticipated clinical judgments, approximately 1/3 of patients that met eligibility criteria of previous positive trials were selected; To these ‘positive controls’, approximately 1/3 of ‘grey zone’ patients with large vessel occlusions, excluded from previous trials (such as patients > 80, patients with minor symptoms, or with a large infarct on imaging), and approximately 1/3 of patients for whom thrombectomy was thought not to be indicated (or negative controls) were added. The clinical information provided for each patient was limited to that which is routinely transmitted between services for making an urgent decision to transfer the patient to a thrombectomy center: age, gender, time of symptom onset, severity of neurological symptoms and signs according to the National Institute of Health Stroke Scale. Key magnetic resonance images of the brain were also provided for each patient. An example of a case from the portfolio and the accompanying questions is presented in Fig. 2.

Thirty-five French stroke centers were randomly selected and offered to participate in the study: 60 neurologists who routinely manage acute ischemic stroke patients and 26 interventional neuroradiologists who routinely perform thrombectomy accepted to participate. For each patient, clinicians were asked 2 questions: 1. “Do you perform/refer this patient for thrombectomy?” (Yes/No); 2.” Would you propose a trial comparing standard therapy with or without thrombectomy for this patient?” (Yes/No) [29].

**Fig. 3:**
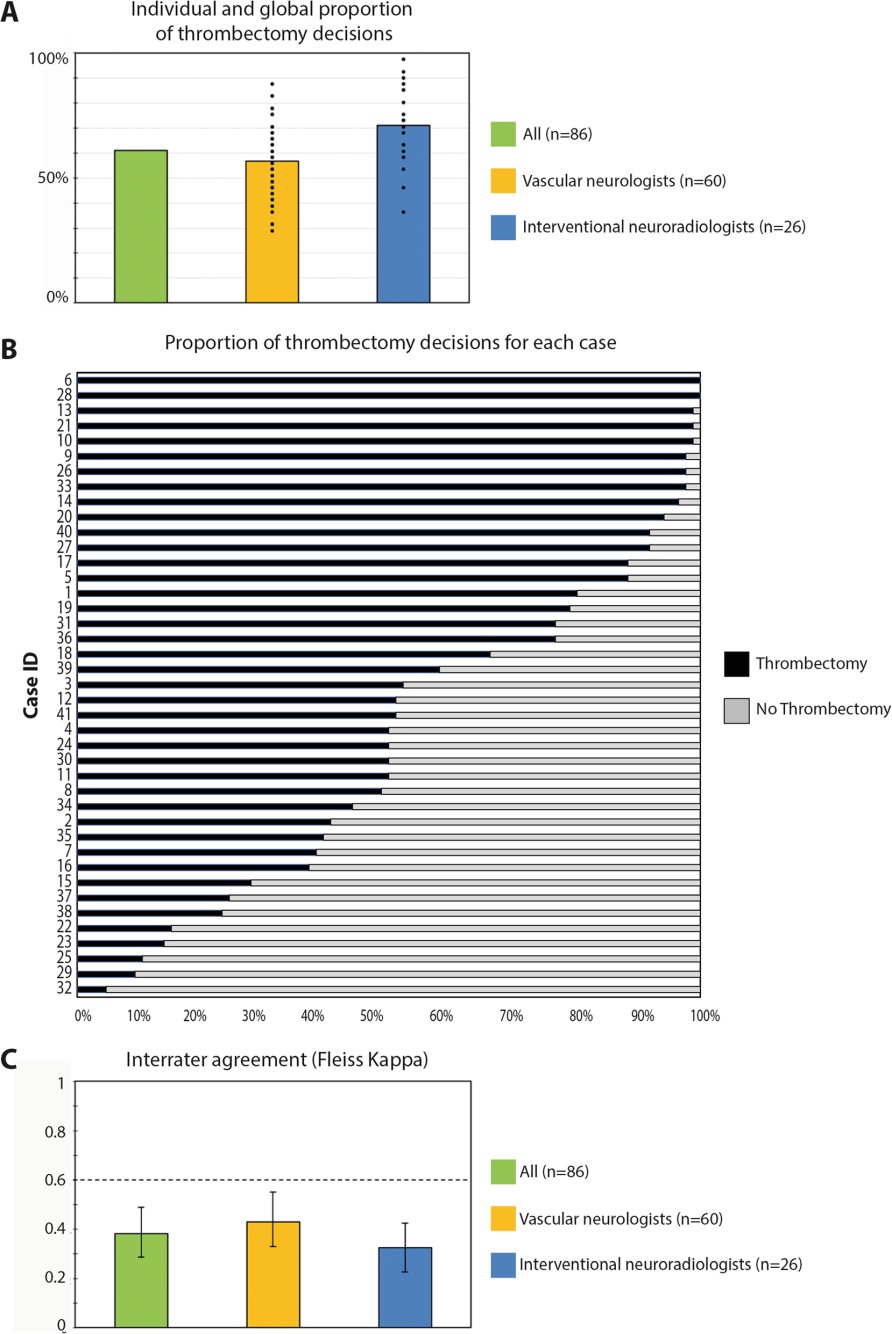
Thrombectomy decisions. Legend: Panel **a** shows the proportion (%) of decisions to perform thrombectomy (in percentages) for all raters and among each specialty. Black dots represent the individual results of each of the 86 clinicians. The bar graphs show similar proportions of decisions between neurologists and interventional neuroradiologists (INRs), but they hide individual discrepancies among physicians, shown here by black dots, revealing a wide range of decisions. Panel **b** shows, for each patient, the proportions (%) of thrombectomy decisions. This panel better illustrates the spectrum of results in various patients, as it shows that some cases had almost unanimous decisions for (complete/almost complete blue bar at the top) or against thrombectomy (complete/almost complete red bar at the bottom part). However, a significant proportion of cases (in the middle) reveal wide disagreements. None of these panels can give an overall idea of the degree of agreement in the study. Panel **c** shows the levels of agreements (through kappa values) in a bar graph. It shows that thrombectomy decisions lack reliability (i.e kappa value is below 0.6) for all raters and also within each subspecialty (vascular neurologists and interventional neuroradiologists)

The total number of thrombectomy decisions was found to vary greatly among neurologists (between 30 and 90%) and among interventional neuroradiologists (between 37 and 98% of decisions). (Fig. 3a). These numbers signal variability, but they are not sufficient to reveal the uncertainty regarding the care of individual patients. It is possible to show the distribution of decisions for each case: Fig. 3b is a bar graph showing the proportions of ‘thrombectomy votes’ for each patient. If for a few patients (at the top and bottom parts of the graph) a majority of raters agreed on whether or not to perform thrombectomy, there was wide disagreement for most patients displayed in between. A summary index of the inter-rater reliability of performing thrombectomy can be calculated using Fleiss’ kappa; values for neurologists and interventionists were well below the ‘substantial’ level, defined as 0.6 by Landis and Koch [35]. (Fig. 3c). Results can also be summarized by observing that at least 1/3 of physicians disagreed on thrombectomy decisions in more than 1/3 of cases. It was concluded there was sufficient clinical uncertainty to conduct additional trials [31].

## Methods

We will follow an order inspired from the Guidelines for Reporting Reliability and Agreement Studies (GRRAS) originally designed for diagnosis/score/measurements [36]. The method presupposes that a dilemma concerning the use of at least 2 different options in the management of some patients affected with a similar clinical problem has previously been identified and will be the object of a randomized trial. It also presupposes that investigators have previously reviewed the pertinent literature, and more specifically the trials that have already been conducted to address the uncertainty, and the remaining concerns and controversies that persist regarding the management of these patients. This normally requires a systematic review. Whether the study is a prelude to a randomized trial or not, investigators need to explicitly identify the clinical problem they are addressing, the spectrum of patients that will be included, the kind of clinicians who will be asked to participate, and the particular clinical management options that will studied.

Two preliminary remarks are in order: first, it is important to mark the difference with a survey of opinions of preferred treatments: More than surveying whether clinicians agree in principle or in theory, regarding certain types of cases in a generic sense, [18, 37] a reliability study is an empirical investigation that tests the reproducibility of judgments made in practice for a series of real particular cases.

Second, real clinical decisions are made once and then acted upon, while studies which assess reproducibility require the independent repetition of the same question concerning the same patient (or the same sort of patients) more than once. Although the study involves real clinicians and real patients, the context of the study is artificial, for decisions do not affect patient care. The experimental set up can be made to somewhat resemble clinical practice, however this may not always be possible, or even desirable (as we will see with the problem of prevalence below).

Investigators are interested in assessing the repeatability of clinical management decisions within and between clinicians on particular patients (Fig. 1). Thus there are 3 components (3 dimensions) to each decision: Each decision D is one of Y choices which are made by one clinician X on one patient Z. Each component (Y, X, or Z) is determined by the experimental design (detailed in the methodology section of the report): 1) Decisions are one of a pre-specified number of categories (spectrum of ‘management choices’, which corresponds to the diagnostic categories of diagnostic studies); 2) Decisions concern an individual belonging to a heterogeneous collection of particular patients affected by the same problem or disease under investigation (spectrum of patients); and 3) Decisions are repeatedly made by a single (intra-rater) or multiple (inter-rater) clinicians of various backgrounds, practices and expertise (spectrum of raters or clinicians). The study team chooses the management categories (the subject of the clinical dilemma) that will be offered as choices and they assemble a collection of patients and of clinician responders. While each decision, clinician and patient are unique, the study must compare decisions to evaluate and summarize their repeatability when they concern the same individual. The agreement study involves preparing a portfolio of patients that is then independently submitted to several physicians. The severity of the reliability test, the subsequent interpretation and the future generalizability of the results all depend on the number and variety of individuals included in the experiment.

### Spectrum of patients

What kind of patients should be included in the study? The classic method to select patients that has the theoretical advantage of allowing statistical inference from the selected individuals to a population is to proceed with random sampling from that population. However, such populations are rarely available in reality. Furthermore, for pragmatic reasons, the number of patients to be studied must be limited, and a small ‘representative’ sample may not include the types and proportions of patients that are necessary to properly test reliability (of diagnoses or of management decisions). Attempts by the study team to duplicate the frequencies naturally found in medical practice in their constructed portfolio can create serious imbalance in the answers obtained. The statistical indices that will be used to summarize results are sensitive to prevalence (or frequency of decisions) [38, 39]. If the object of the diagnostic reliability study is a rare disease, for example, the portfolio cannot include the proportion of patients naturally affected (say 1/1000); the same goes for management categories (such as invasive surgery). Finally, we must remember that we are not interested in capturing an index which estimates the distribution of a disease or characteristic in a population, nor in finding out which management option is most frequently used by a population of doctors, but the goal of the study is to rigorously test whether the clinical judgments that are made are repeatable, one patient at a time, no matter the circumstances, clinicians or patients. Thus, while the portfolio must include a diversity of patients, and it may be constructed to resemble a clinical series, it does not have to be ‘representative’ of a theoretical population of patients. The challenge is more akin to testing an experimental apparatus in a laboratory with specimens of a known composition (positive and negative controls), prior to using the apparatus to explore specimens of unknown composition. Just as the reliability of a balance is not rigorously tested by weighing the same object 10 times, or by weighing objects of very similar weights, but by testing it with a wide range of weights, the reliability of clinical judgments must be tested with a diversity of particular patients, ideally covering a wide range of possible clinical encounters, along various spectra (age, size, location, duration of symptoms etc..), whether they concern diagnostic verdicts or therapeutic decisions. In practice, the portfolio will typically be artificially constructed to include prototypical patients selected by members of the study team (who are familiar with the clinical dilemma) to be ‘positive’ and ‘negative controls’ for the various diagnostic or decision categories, to make sure they will be represented in the final decisions, as well as a substantial proportion of less typical or ‘grey zone’ cases.

The amount of information which should be provided for each patient included in the portfolio is a difficult question. To minimize the chance that clinicians might disagree based simply on different interpretation of the information provided, we believe it should be limited to the essential, for the purpose of the study is not to identify all potential reasons to disagree on a particular patient, but to measure the clinical uncertainty that remains even when extraneous reasons for potential disagreement are minimized.

While each patient included in the study is a concrete particular, sometimes uniquely identified by their radiograph or angiogram, for example, [22, 23, 25] the patient can always be grouped (at the time of clinical decisions or at the time of analyses) with other patients in a number of conceptual generalizations (or subgroups) that, according to some background knowledge pertinent to the clinical dilemma being studied, can influence clinical decisions. Investigators may be interested in exploring which patient or disease characteristic is associated with which decision. Patient or disease characteristics that will be included in each particular clinical vignette of the portfolio are generalizations (sometimes each with its own spectrum) that may influence decisions. These may or may not be ‘reasons for decisions’ or ‘reasons for actions’, and they may be weighted differently by different clinicians. Investigators interested in exploring such details should ensure they include a sufficient number of particular patients with and without the characteristics of interest in the portfolio.

Like the baseline characteristics included in the registration form of a clinical trial, the information must be made available for each patient and expressed in a standardized fashion. These baseline characteristics are summarized in a descriptive Table of patients included in the study.

The source of patients included in the study should be mentioned in the study report. Patients may be selected from the data base of a registry or of a clinical trial. In such cases, the selection criteria of the trial should be mentioned. The exact selection of cases will of course impact results; the series of cases can be provided *in extensio* at the time of publication.

### Spectrum of clinicians

The study of the reliability of clinical decisions should include numerous clinicians of various backgrounds and experiences, from all specialties involved in the various management options pertinent to the dilemma under study, as each specialty shares a body of knowledge and beliefs (and frequently a preference for the treatment it usually performs). What renders a scale or a treatment recommendation reliable, is that judgments are repeatable even when made by clinicians of various backgrounds and experience in diverse patients. The questionnaire will collect some baseline information on participating clinicians, and the characteristics of the clinicians involved in the study can be detailed in a table. Results can also be analyzed separately for some subgroups of clinicians (for each specialty, or for experienced or ‘senior’ clinicians). Of course, clinicians from various specialties may have diverging opinions, but even colleagues with the same background working in the same center and exposed to similar experiences may not make the same treatment recommendation for the same patient [22, 31]. The goal of the study is not to find which treatment is most popular in some population of specialists, nor to try to identify ‘the right treatment’ by polling opinions. Thus it is not necessary for clinicians to be a representative sample of one specialty or another (although they may be). Participants responding to the survey are asked to seriously consider each case as if it were a momentous clinical decision, but respondents should be reassured they will not be judged; they should not be afraid of being “wrong”, because unlike an accuracy study, there is no gold standard with which to evaluate performance.

The problem is more delicate with intra-rater studies. These may be very informative, but they are rarely performed [31, 40, 41]. Better agreement can be expected when the same clinician responds twice to the same series of cases (typically weeks apart in patients presented in a different order to assure independence between judgments), but the risk here is that the clinician may reveal their own inconsistencies in decision-making. In the case of diagnostic tests, poor intra-rater agreement (across multiple raters) is evidence of the lack of reliability of the score/measurement/diagnostic categories, and a strong indication that the scale or categories should be modified [40]. We see no reason to conclude differently with management decisions: when asked the same question twice, a clinician’s inconsistencies in recommending opposing options to the same patient only reasserts a high degree of uncertainty regarding the clinical dilemma being examined. Participating in such intra-rater studies can be a humbling experience, but one that can convince the participant that a clinical trial may be in order.

### Management categories

For each case, clinicians are independently asked which predefined option they would recommend or carry out. Choices are readily made when the questionnaire is conceived at the time of the design of a randomized controlled trial (RCT): the options are the 2 treatments being compared. Particular attention should be paid to the wording of questions, as ambiguities can affect the reliability of responses. Of course, agreement will be less frequent when the number of possible options is increased: it is more difficult to agree on the use of various treatments (“would you use A, B, C, or conservative management?”), than agreeing on: “would you treat this patient with A? [Yes/No].” Categorical responses can sometimes be dichotomized at the time of analyses [22, 25].

Results will of course depend on the way the questions are formulated, and the best way to conceive the questionnaire will depend on the particular object of the study.

The questionnaire may be given a test run with a few ‘test patients’ on a few ‘test clinicians’ before proceeding with the real study, as the wording of the questions included may need to be modified when problems with the first iterations are encountered.

### Additional questions

The investigators may ask, for each decision, the level of confidence of participants [22, 23, 25]. If the questionnaire is prepared as a preliminary step in the design of a RCT, participants can also be asked the direct question: would you propose, to this patient, participation in a trial that randomly allocates treatments A and B? [22, 24, 25, 31].

### Statistical power and analyses

The number of cases and clinicians necessary to judge reliability with sufficient rigor and power depends on several parameters [42].

This number, predefined and justified in the study protocol, should be large enough to ensure the study can provide estimates of reliability that are precise enough (confidence intervals narrow enough) to be meaningful.

The number of patients to be studied is typically limited for pragmatic reasons. The larger the number of cases to be studied, the smaller the number of clinicians willing to participate. We have found that for simple questions with a binary outcome, as a rule of thumb a minimal number of ten raters reviewing 30–50 patients is necessary for the study to be informative [23–25, 40].

There are many statistical approaches to measure reliability and agreement, depending on the type of data (categorical, ordinal, continuous), the sampling method and on the treatment of errors [36]. Reliability in treatment recommendations (categories) is most frequently analyzed using kappa-like statistics. There are several types of kappa statistics, and a discussion of the appropriate use of one or the other is beyond the scope of this article. A statistician should be involved in the design of the study early on.

Analyses can be repeated for various subgroups of patients or clinicians. For example, in the case of an agreement study involving physicians from various specialties, it can be useful to study the degree of agreement within each specialty, to show that disagreements are not explained by various training or backgrounds [25, 31].

Similarly, if it is known that some patient characteristic is commonly used to select one option rather than the other, agreement for patients sharing that characteristic can be analyzed. It should be noted that subgroup analyses reduce the number of observations; there may not be a sufficient variety and number of cases to adequately assess the reliability of decisions regarding that particular characteristic; confidence intervals are irremediably wider and results should be interpreted with caution.

## Reporting results

The report should be transparent and follow standardized guidelines (Table 1) [36].

**Table 1:** Reporting patient management agreement studies (inspired from GRRAS)

**TITLE AND ABSTRACT**	1. Identify in title and/or abstract the clinical dilemma for which uncertainty and intra-inter physician agreement was investigated
**INTRODUCTION**	2. Name and describe the subject of interest explicitly: what disease(s), what available management options, what clinical dilemmas are being considered
	3. Specify the patients that are confronted with uncertainty
	4. Specify the clinicians involved in making clinical decisions or recommendations
	5. Describe what is already known about reliability/agreement and provide a rationale for the study.
**METHODS**	6. Explain how the number of patients and clinicians was chosen.
	7. Describe how patients and clinicians were selected.
	8. Describe the experimental setting (e.g time interval between sessions, availability of clinical information, blinding…)
	9. State whether judgments were made independently.
	10. Describe the statistical analyses
**RESULTS**	11. State the actual number of raters and subjects that were included, and the number of replicated judgments which were collected.
	12. Describe the characteristics of clinicians (training, experience) and patients (any clinical data judged relevant to the study question).
	13. Report estimates of reliability and agreement including measures of statistical uncertainty.
**DISCUSSION**	Discuss the practical relevance of results.
**AUXILIARY MATERIAL**	Provide detailed results if possible (e.g online).

The results section normally includes descriptive statistics regarding the total number of management decisions, summarized in tables or figures. Comparing decision categories made by various subgroups of clinicians on various subgroups of patients may sometimes be of interest.

The most important results concerning the repeatability of management decisions are typically expressed using indices (such as kappa values) summarized in tables or figures to allow a rapid appreciation of the overall results and simplify comparisons between subgroups. Unfortunately, such indices often have little meaning to clinicians. While a scale of interpretation can be provided (such as Landis and Koch [35]), interpretation can be facilitated by translating results into clinically meaningful sentences. To use the thrombectomy example: In practical terms, at least 5/20 clinicians (25%) changed their own decision regarding mechanical thrombectomy in 17.1% (7/41) of cases, respectively.” Providing particular examples at both extremes of the spectrum (cases with near-perfect agreement and cases with maximal disagreement, when they occur) may also help illustrate the results of the study.

## Discussion

The studies we propose are designed to identify and measure clinical uncertainty defined as the repeatability of clinical decisions made by particular clinicians on particular patients. While the methodology and statistical apparatus already exist, they have rarely if ever been applied to clinical decisions and interventions, probably because such decisions are held to be singular, unrepeatable, individually made according to clinical judgment, taking into account particular circumstances, personal interests and values [13]. In other words, while a diagnostic category can be tested for its reliability (for it should objectively be re-identifiable), clinical decisions and management options are unanalyzable in that fashion, for they are subjective, value-laden choices that are freely made according to intentions and personal preferences. The widely-held notion that individualized care requires the identification of ‘the right action’ in each patient, if true, would leave no hope to replace unverifiable medical care based on good intentions by verifiable medical care based on patient outcomes. The truth is that for most clinical situations, clinical judgement and informed decisions end up in a limited variety of repeatable categories. The central idea of the methodology we propose is simply to replace a diagnostic category (say, to use our illustrative example, ‘patient with acute stroke’) with a ‘therapeutic category’ (say ‘patient with acute stroke I would treat using thrombectomy’). The standard ways to assess the reliability of allocating a category to an individual patient, and the same statistical indices can then be used to measure clinical uncertainty in the management of individual patients.

The methodology we propose can undoubtedly be improved with experience as other investigators explore the best way to adapt it to other areas of medical care. We have mainly explored the use of this methodology in assessing clinical uncertainty in the surgical or endovascular care of patients with cerebrovascular diseases, such as aneurysms, arteriovenous malformations and stroke. Because many interventions are image-guided or crucially depend on particular anatomical features of the patient’s lesion, we very naturally came to use individual vascular images of particular patients to assess the reliability of clinical decisions [23–25]. How the approach can apply to other medical fields remains to be explored; one difficulty is to find a way to present a variety of individual patients, pertinent to the clinical dilemmas that would be examined. It is important to distinguish what we here propose from other research programs: we are not trying to identify local practice variations [43], we are not polling expert opinions [37]; we are not studying medical decision-making (and associated problems such as framing effects) [44–46]; we are not trying to improve decision quality [47]. The studies we have so far performed have been very simple [22–25]. We have not attempted to identify reasons for disagreements, for example by repeating the assessment of the same patients, but with some modification or other of some feature of the particular history. Identifying ‘reasons to prefer this option’, or membership in one subgroup or other, is in the end adding or multiplying intermediate categories between diagnoses and interventions. There are just too many ‘reasons’ to choose. These new categories would in turn need to be studied for their reliability, leading to an infinite regress or an explosion of studies. In our view, trying to explain disagreements is misguided, for most of the time we know why there is uncertainty and disagreement: no one knows which option is best, for reliable studies comparing the outcomes of patients treated differently have not been conducted.

The recognition and estimation of clinical uncertainties could serve many purposes: first, we believe the clinical community, clinicians and patients alike, should be aware that diverse options are actually being proposed for the management of similar patients, if only to make alternative options available. Second, recognizing the uncertainty may be the first important step towards a true science of medical practice, for this step may encourage members of the community to get organized and prepare for the work that needs to be done: to accept the uncertainty revealed by the study, and proceed with the clinical research that addresses that uncertainty. But not any type of research will do.

Trying to side-step the uncertainty and persistently trying to identify ‘the right action’ through computerized decisions aids [48] or ‘shared-decision making’, when treatment outcomes have not reliably been verified, has been shown to be dangerous. A notable example was when a decision aid misled patients into choosing between cardioprotection and breast cancer risks with menopausal hormonal therapy, while such therapy was later shown to increase both cardiovascular and cancer risks when proper trials were finally conducted [49].

If the first step of a science of practice is to recognize the uncertainty, the second step is to change practice to take into account that uncertainty. Thus the studies we propose can be a prelude to the design or conduct of care trials, which are pragmatic trials integrated into care, designed in the best medical interest of the patient, with no extra test, risk, or cost [4, 27, 28, 30]. When a reliability study is designed with a trial in view, it can provide empirical evidence of Freedman’s notion of ‘clinical equipoise’, or professional disagreement among expert clinicians about the preferred treatment, [6] a result that may reassure clinicians, patients and ethics committees. While in our view no such equipoise condition is necessary when evidence regarding what to do is lacking, randomized allocation to 2 different options may become impossible if all clinicians agree on one option for a particular group of patients (an unlikely event). The range of kappa values could be interpreted within a scale of uncertainty that could indicate the likelihood of recruitment, as suggested in Table 2. Of course, the predictive value of any such scale on the recruitment actually achieved would need to be empirically verified.

**Table 2:**
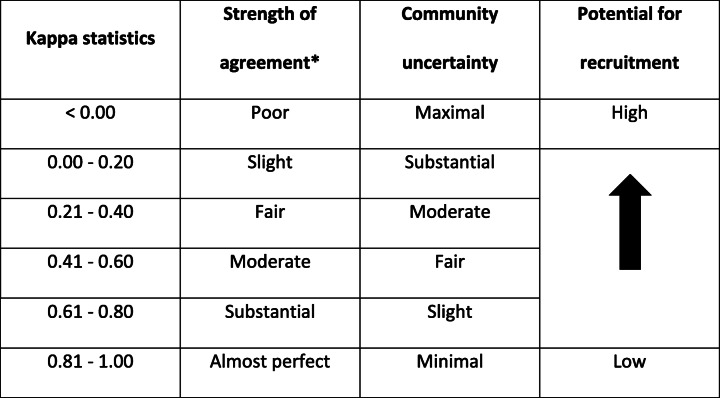
Index of uncertainty and potential for trial recruitment

*According to Landis and Koch. [35]

RCTs remain poorly accepted by patients and the medical community alike [10, 50–52]. One obstacle is the notion that by participating in a RCT, the clinician abdicates and the patient exchanges a personalized decision for randomized allocation, a method whose sole purpose is to decrease bias and provide generalizable knowledge. This idea ignores the benefits randomized allocation can play in balancing the risks of receiving an inferior treatment [53, 54]. If medical care should always be individualized, this does not mean that the doctor always knows what to do. But the notion that the ‘right action’ needs to be identified to provide personalized care has encouraged dogmatic, unverifiable medical practices. We believe patients confronted with such dilemmas, as established by low agreement in these types of studies, are better cared for within the context of a care trial. The fact that equally respected clinicians would have chosen the rival treatment option could reassure clinicians and patients that the treatment that is randomly allocated is a treatment the patient could have received had they sought the opinion of a different expert.

## Conclusion

Reliability studies of clinicians’ recommendations can reveal and measure clinical uncertainty regarding the best treatment, increase open-mindedness regarding the possibility of alternative options, provide an empirical foundation for the notion of equipoise, and inform or facilitate the design/conduct of clinical trials to address the clinical dilemma. Such studies may show the necessity to change the way we practice, from unrepeatable, unverifiable decisions, to a more prudent and systematic approach that takes uncertainty into account. When no one really knows what to do, integrating research methods to clinical care may be in the best medical interest of individual patients. Collecting empirical evidence regarding variability in treatment recommendations may, in the future, become an important component of a science of clinical practice.

## Data Availability

NA

## Abbreviations

GRRAS: Guidelines for reporting reliability and agreement studies
NIHSS: National institute of health stroke scale
RCT: Randomized controlled trial

## References

[1] Ford I, Norrie J (2016). Pragmatic trials. N Engl J Med.

[2] Simon GE, Platt R, Hernandez AF (2020). Evidence from pragmatic trials during routine care - slouching toward a learning health system. N Engl J Med.

[3] Thabane L, Kaczorowski J, Dolovich L, Chambers LW, Mbuagbaw L (2015). CHAP investigators. Reducing the confusion and controversies around pragmatic trials: using the cardiovascular health awareness program (CHAP) trial as an illustrative example. Trials.

[4] Raymond J, Darsaut TE, Altman DG (2014). Pragmatic trials used as optimal medical care: principles and methods of care trials. J Clin Epidemiol.

[5] Fried C (2016). Medical experimentation: personal integrity and social policy: new edition.

[6] Freedman B (1987). Equipoise and the ethics of clinical research. N Engl J Med.

[7] Horn AR, Weijer C, Grimshaw J, Brehaut J, Fergusson D, Goldstein CE, Taljaard M (2018). An ethical analysis of the SUPPORT trial: addressing challenges posed by a pragmatic comparative effectiveness randomized controlled trial. Kennedy Inst Ethics J.

[8] Joffe S, Miller FG (2008). Bench to bedside: mapping the moral terrain of clinical research. Hast Cent Rep.

[9] Lantos JD (2014). Learning the right lessons from the SUPPORT study controversy. Arch Dis Child Fetal Neonatal Ed.

[10] Kelley M, James C, Alessi Kraft S, Korngiebel D, Wijangco I, Rosenthal E, Joffe S, Cho MK, Wilfond B, Lee SS (2015). Patient perspectives on the learning health system: The importance of trust and shared decision making. Am J Bioeth.

[11] Mark DB, Wong JB, Hauser S, Longo D, Jameson J, Loscalzo J (2014). Decision making in clinical medicine. Harrison's principal of internal medicine.

[12] Walicke P, Abosch A, Asher A, Barker FG, Ghogawala Z, Harbaugh R, Jehi L, Kestle J, Koroshetz W, Little R, Rubin D, Valadka A, Wisniewski S, Chiocca EA, For The Workshop Participants (2017). Launching effectiveness research to guide practice in neurosurgery: a national institute neurological disorders and stroke workshop report. Neurosurgery.

[13] The PE, Engelhardt HT, Spicker SFBT (1977). Anatomy of Clinical Judgments. Clinical Judgment: A Critical Appraisal Philosophy and Medicine.

[14] Braddock CH, Edwards KA, Hasenberg NM, Laidley TL, Levinson W (1999). Informed decision making in outpatient practice: time to get back to basics. J Am Med Assoc.

[15] Legare F, Moher D, Elwyn G, LeBlanc A, Gravel K (2007). Instruments to assess the perception of physicians in the decision-making process of specific clinical encounters: a systematic review. BMC Med Inform Decis Mak.

[16] O'Connor AM, Stacey D, Entwistle V, Llewellyn-Thomas H, Rovner D, Holmes-Rovner M, Tait V, Tetroe J, Fiset V, Barry M, Jones J (2003). Decision aids for people facing health treatment or screening decisions. Cochrane Database Syst Rev.

[17] Bankier AA, Levine D, Halpern EF, Kressel HY (2010). Consensus interpretation in imaging research: is there a better way?. Radiology.

[18] Etminan N, Brown RD, Beseoglu K, Juvela S, Raymond J, Morita A, Torner JC, Derdeyn CP, Raabe A, Mocco J, Korja M, Abdulazim A, Amin-Hanjani S, Al-Shahi Salman R, Barrow DL, Bederson J, Bonafe A, Dumont AS, Fiorella DJ, Gruber A, Hankey GJ, Hasan DM, Hoh BL, Jabbour P, Kasuya H, Kelly ME, Kirkpatrick PJ, Knuckey N, Koivisto T, Krings T, Lawton MT, Marotta TR, Mayer SA, Mee E, Pereira VM, Molyneux A, Morgan MK, Mori K, Murayama Y, Nagahiro S, Nakayama N, Niemelä M, Ogilvy CS, Pierot L, Rabinstein AA, Roos YB, Rinne J, Rosenwasser RH, Ronkainen A, Schaller K, Seifert V, Solomon RA, Spears J, Steiger HJ, Vergouwen MD, Wanke I, Wermer MJ, Wong GK, Wong JH, Zipfel GJ, Connolly ES, Steinmetz H, Lanzino G, Pasqualin A, Rüfenacht D, Vajkoczy P, Mcdougall C, Hänggi D, LeRoux P, Rinkel GJ, Macdonald RL (2015). The unruptured intracranial aneurysm treatment score: a multidisciplinary consensus. Neurology.

[19] Fahed R, Darsaut TE (2017). The Delphi Oracle and the management of aneurysms. J Neurointerv Surg.

[20] Cenzato M, Boccardi E, Beghi E, Vajkoczy P, Szikora I, Motti E, Regli L, Raabe A, Eliava S, Gruber A, Meling TR, Niemela M, Pasqualin A, Golanov A, Karlsson B, Kemeny A, Liscak R, Lippitz B, Radatz M, La Camera A, Chapot R, Islak C, Spelle L, Debernardi A, Agostoni E, Revay M, Morgan MK (2017). European consensus conference on unruptured brain AVMs treatment (supported by EANS, ESMINT, EGKS, and SINCH). Acta Neurochir.

[21] Magro E, Gentric JC, Darsaut TE, Raymond J, the TOBASinvestigators (2017). Unruptured brain AVMs: it's time we worked together to integrate care and clinical research. Acta Neurochir.

[22] Darsaut TE, Estrade L, Jamali S, Bojanowski MW, Chagnon M, Raymond J (2014). Uncertainty and agreement in the management of unruptured intracranial aneurysms. J Neurosurg.

[23] Darsaut TE, Fahed R, Macdonald RL, Arthur AS, Kalani MYS, Arikan F, Roy D, Weill A, Bilocq A, Rempel JL, Chow MM, Ashforth RA, Findlay JM, Castro-Afonso LH, Chagnon M, Gevry G, Raymond J (2018). Surgical or endovascular management of ruptured intracranial aneurysms: an agreement study. J Neurosurg.

[24] Darsaut TE, Gentric JC, McDougall CM, Gevry G, Roy D, Weill A, Raymond J (2015). Uncertainty and agreement regarding the role of flow diversion in the management of difficult aneurysms. Am J Neuroradiol.

[25] Fahed R, Batista AL, Darsaut TE, Gentric JC, Ducroux C, Chaalala C, Roberge D, Bojanowski MW, Weill A, Roy D, Magro E, Raymond J (2017). The treatment of brain Arteriovenous malformation study (TOBAS): a preliminary inter- and intra-rater agreement study on patient management. J Neuroradiol.

[26] Darsaut TE, Findlay JM, Magro E, Kotowski M, Roy D, Weill A, Bojanowski MW, Chaalala C, Iancu D, Lesiuk H, Sinclair J, Scholtes F, Martin D, Chow MM, O'Kelly CJ, Wong JH, Butcher K, Fox AJ, Arthur AS, Guilbert F, Tian T, Chagnon M, Nolet S, Gevry G, Raymond J (2017). Surgical clipping or endovascular coiling for unruptured intracranial aneurysms: a pragmatic randomised trial. J Neurol Neurosurg Psychiatry.

[27] Darsaut TE, Jack AS, Kerr RS, Raymond J (2013). International subarachnoid aneurysm trial - ISAT part II: study protocol for a randomized controlled trial. Trials.

[28] Darsaut TE, Magro E, Gentric JC, Batista AL, Chaalala C, Roberge D, Bojanowski MW, Weill A, Roy D, Raymond J (2015). Treatment of brain AVMs (TOBAS): study protocol for a pragmatic randomized controlled trial. Trials.

[29] Khoury NN, Darsaut TE, Ghostine J, Deschaintre Y, Daneault N, Durocher A, Lanthier S, Pope AY, Odier C, Lebrun LH, Guilbert F, Gentric JC, Batista A, Weill A, Roy D, Bracard S, Raymond J, EASI trial collaborators (2017). Endovascular thrombectomy and medical therapy versus medical therapy alone in acute stroke: a randomized care trial. J Neuroradiol.

[30] Raymond J, Gentric JC, Darsaut TE, Iancu D, Chagnon M, Weill A, Roy D (2017). Flow diversion in the treatment of aneurysms: a randomized care trial and registry. J Neurosurg.

[31] Ducroux C, Fahed R, Khoury N, Gevry G, Kalsoum E, Labeyrie M, Ziegler D, Sauve C, Changnon M, Darsaut T, Raymond J, FAMOUS collaborative group (2019). Intravenous thrombolysis and thrombectomy decisions in acute ischemic stroke: an interrater and intrarater agreement study. Rev Neurol (Paris).

[32] Berkhemer OA, Fransen PS, Beumer D, van den Berg LA, Lingsma HF, Yoo AJ, Schonewille WJ, Vos JA, Nederkoorn PJ, Wermer MJ, van MAA W, Staals J, Hofmeijer J, van Oostayen JA, Lycklama à Nijeholt GJ, Boiten J, Brouwer PA, Emmer BJ, de Bruijn SF, van Dijk LC, Kappelle LG, Lo RH, van Dijk EJ, de Vries J, de Kort PLM, van Rooij WJJ, van den Berg JSP, van Hasselt BAAM, LAM A, Dallinga RJ, Visser MC, JCJ B, Vroomen PC, Eshghi O, THCML S, RJJ H, Keizer K, Tielbeek AV, den Hertog HM, Gerrits DG, van den Berg-Vos RM, Karas GB, Steyerberg EW, Flach HZ, Marquering HA, MES S, SFM J, LFM B, van den Berg R, Koudstaal PJ, van Zwam WH, YBWEM R, van der Lugt A, van Oostenbrugge RJ, CBLM M, DWJ D, MR CLEAN Investigators (2015). A randomized trial of intraarterial treatment for acute ischemic stroke. N Engl J Med.

[33] Campbell BC, Mitchell PJ, Kleinig TJ, Dewey HM, Churilov L, Yassi N, Yan B, Dowling RJ, Parsons MW, Oxley TJ, Wu TY, Brooks M, Simpson MA, Miteff F, Levi CR, Krause M, Harrington TJ, Faulder KC, Steinfort BS, Priglinger M, Ang T, Scroop R, Barber PA, McGuinness B, Wijeratne T, Phan TG, Chong W, Chandra RV, Bladin CF, Badve M, Rice H, de Villiers L, Ma H, Desmond PM, Donnan GA, Davis SM, EXTEND-IA Investigators (2015). Endovascular therapy for ischemic stroke with perfusion-imaging selection. N Engl J Med.

[34] Raymond J, Fahed R, Roy D, Darsaut TE (2019). The 2018 ter Brugge lecture: problems with the Introduction of innovations in neurovascular care. Can J Neurol Sci.

[35] Landis JR, Koch GG (1977). The measurement of observer agreement for categorical data. Biometrics.

[36] Kottner J, Audigé L, Brorson S, Donner A, Gajewski BJ, Hróbjartsson A, Roberts C, Shoukri M, Streiner DL (2011). Guidelines for reporting reliability and agreement studies (GRRAS) were proposed. J Clin Epidemiol.

[37] Cockroft KM, Chang KE, Lehman EB, Harbaugh RE (2014). AVM management equipoise survey: physician opinions regarding the management of brain arteriovenous malformations. J Neurointerv Surg.

[38] Cicchetti DV, Feinstein AR. High agreement but low kappa: II. Resolving the paradoxes J Clin Epidemiol 1990, 43:551–558. 10.1016/0895-4356(90)90159-m 2189948

[39] Feinstein AR, Cicchetti DV (1990). High agreement but low kappa: I. The problems of two paradoxes. J Clin Epidemiol.

[40] Farzin B, Fahed R, Guilbert F, Poppe AY, Daneault N, Durocher AP, Lanthier S, Boudjani H, Khoury NN, Roy D, Weill A, Gentric JC, Batista A, Létourneau-Guillon L, Bergeron F, Henry MA, Darsaut TE, Raymond J (2016). Early CT changes in patients admitted for thrombectomy: Intrarater and interrater agreement. Neurology.

[41] Farzin B, Gentric JC, Pham M, Tremblay-Paquet S, Brosseau L, Roy C, Jamali S, Chagnon M, Darsaut TE, Guilbert F, Naggara O, Raymond J (2017). Agreement studies in radiology research. Diagn Interv Imaging.

[42] Donner A, Rotondi MA. Sample size requirements for interval estimation of the kappa statistic for interobserver agreement studies with a binary outcome and multiple raters. Int J Biostat. 2010;6(1):Article 31. 10.2202/1557-4679. 10.2202/1557-4679.1275 21969984

[43] Wennberg J, Gittelsohn A. Small area variations in health care delivery. Science. 1973;182:1102–8.. 10.1126/science.182.4117.1102 4750608

[44] Bernstein LM, Chapman GB, Elstein AS (1999). Framing effects in choices between multi-outcome life-expectancy lotteries. Med Decis Mak.

[45] Mishra S, Gregson M, Lalumiere ML (2012). Framing effects and risk-sensitive decision making. Br J Psychol.

[46] Poses RM, Krueger JI, Sloman S, Elstein AS (2002). Physicians' judgments of survival after medical management and mortality risk reduction due to revascularization procedures for patients with coronary artery disease. Chest.

[47] Sepucha KR, Fowler Jr FJ, Mulley Jr AG. Policy support for patient-centered care: the need for measurable improvements in decision quality. Health Aff (Millwood). 2004, Suppl Variation:VAR54–62. 10.1377/hlthaff.var.54. 10.1377/hlthaff.var.54 15471772

[48] Parmigiani G (2002). Measuring uncertainty in complex decision analysis models. Stat Methods Med Res.

[49] Col NF, Ngo L, Fortin JM, Goldberg RJ, O'Connor AM (2007). Can computerized decision support help patients make complex treatment decisions? A randomized controlled trial of an individualized menopause decision aid. Med Decis Mak.

[50] Fiorella D, Mocco J, Arthur A, Siddiqui A, Heck D, Albuquerque F, Turk A (2015). Randomized controlled trials for everything?. J Neurointerv Surg.

[51] Mansouri A, Cooper B, Shin SM, Kondziolka D (2016). Randomized controlled trials and neurosurgery: the ideal fit or should alternative methodologies be considered?. J Neurosurg.

[52] Robinson EJ, Kerr CE, Stevens AJ, Lilford RJ, Braunholtz DA, Edwards SJ, Beck SR, Rowley MG (2005). Lay public's understanding of equipoise and randomisation in randomised controlled trials. Health Technol Assess.

[53] Fahed R, Darsaut TE, Raymond J (2018). The Introduction of innovations in neurovascular care: patient selection and randomized allocation. World Neurosurg.

[54] Raymond J, Fahed R, Darsaut TE (2017). Randomize the first patient. J Neuroradiol.

